# Sustainable Nanomaterials for Biomedical Applications

**DOI:** 10.3390/pharmaceutics15030922

**Published:** 2023-03-12

**Authors:** Yuhang Zhang, Kingsley Poon, Gweneth Sofia P. Masonsong, Yogambha Ramaswamy, Gurvinder Singh

**Affiliations:** 1 School of Biomedical Engineering, The University of Sydney, Camperdown, NSW 2008, Australia; 2 Sydney Nano Institute, The University of Sydney, Camperdown, NSW 2008, Australia

**Keywords:** nanotechnology, sustainability, functional nanomaterials, renewable resources, green synthesis, circular economy, biomedical, sustainable materials design

## Abstract

Significant progress in nanotechnology has enormously contributed to the design and development of innovative products that have transformed societal challenges related to energy, information technology, the environment, and health. A large portion of the nanomaterials developed for such applications is currently highly dependent on energy-intensive manufacturing processes and non-renewable resources. In addition, there is a considerable lag between the rapid growth in the innovation/discovery of such unsustainable nanomaterials and their effects on the environment, human health, and climate in the long term. Therefore, there is an urgent need to design nanomaterials sustainably using renewable and natural resources with minimal impact on society. Integrating sustainability with nanotechnology can support the manufacturing of sustainable nanomaterials with optimized performance. This short review discusses challenges and a framework for designing high-performance sustainable nanomaterials. We briefly summarize the recent advances in producing sustainable nanomaterials from sustainable and natural resources and their use for various biomedical applications such as biosensing, bioimaging, drug delivery, and tissue engineering. Additionally, we provide future perspectives into the design guidelines for fabricating high-performance sustainable nanomaterials for medical applications.

## 1. Introduction

The world population has rapidly increased from 7 billion to 8 billion in the last decade. This has placed tremendous pressure and a socio-economic burden on a better and more affordable healthcare system to protect people from infectious and life-threatening diseases. Additionally, the world is facing grave environmental, climate, and energy challenges. In 2015, the United Nations (UN) established 17 sustainable development goals (SDGs) to address such challenges. These SDGs aim to eradicate poverty, provide better healthcare to various communities, and tackle societal challenges using renewable and sustainable materials. The UN has arguably recognized the role of nanotechnology in achieving 13 out of 17 SDGs by 2030. Nanotechnology has emerged as a game-changing technology for fabricating nanoscale materials. A large surface area to volume and the unique size, shape, and composition-dependent characteristics of nanomaterials make them suitable for various practical applications from biomedical to renewable energy and the environment. In the biomedical field, the scientific revolution of nanotechnology has witnessed the discovery of mRNA vaccines for COVID-19 using lipid nanoparticles [1], the development of wearable medical devices/sensors [2], and wireless bandages stimulating wound healing for people living in both urban and rural regions [3].

**Figure 1:**
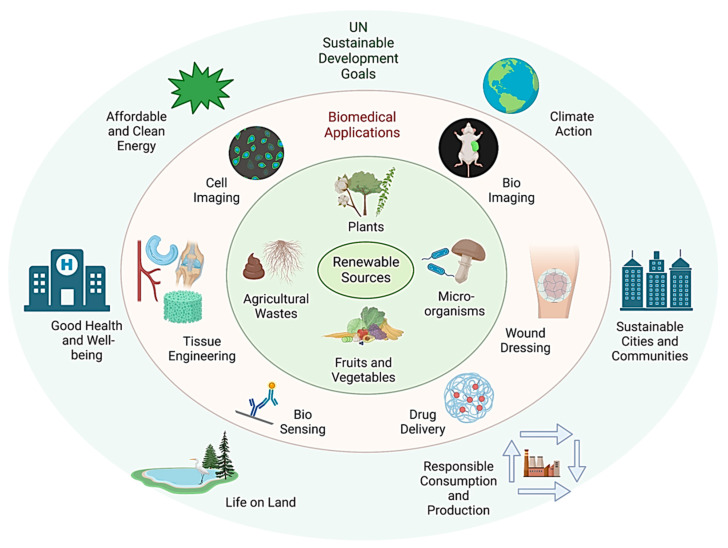
Renewable resources to design sustainable nanomaterials and their biomedical applications for a sustainable future.

Nanotechnology holds great promise for developing the next generation of medical devices and sensors [4,5], implants [6], nanovaccines [7], diagnostics [8,9], and therapeutic technologies (Figure 1) [10,11]. In the last two decades, intensive research attempts have been made to develop manufacturing strategies involving top-down (lithography and etching) and bottom-up (chemical reduction and sol–gel) for designing functional nanomaterials. These manufacturing methods produce hazardous waste as a by-product, posing an immediate risk to workers and the environment. Furthermore, the mismanagement in the synthesis and handling of nanomaterials can cause severe short-term and long-term consequences to human health and the environment [12]. Though targeted nanomaterials-based chemotherapeutic agents have been developed for treating cancerous diseases [13], the long-term consequences of these nanomaterials on human health, such as inflammation, toxicity, the degradation mechanism of the nanomaterials, alteration in the function of organs and subsequent tumor development, or genetic disorders have not been prioritized in the past decade. Moreover, the issues related to safely manufacturing, nanomaterials’ safe handling, and lack of clinical data have predominantly restricted the translation of nanomaterials-based medicine for clinical applications. Therefore, the choice of raw materials (precursor materials), manufacturing method (cost and scalability), and handling protocols of nanomaterials should be considered when designing safe nanomaterials for biomedical applications. Manufacturing nanomaterials from renewable resources using green synthesis principles can enable the design of safe and sustainable nanomaterials.

Sustainable nanotechnology encompasses the safe manufacturing of sustainable nanomaterials during the design phase of product development while minimizing nanomaterials’ impact on society and the environment. In order to set sustainable manufacturing practices, it is imperative to consider the entire life cycle of the product, from the raw materials extraction (primary precursors, surfactants, and non-hazardous solvents from the natural and sustainable resources), to manufacturing methods, and recycling and reuse at the product’s end of life. In recent years, several review articles have been published on synthesizing functional nanomaterials and their biomedical applications, from bioimaging to biosensing, diagnosis, and therapeutics [14,15,16,17]. However, reports on the design of safe and sustainable nanomaterials are scarce in the literature. Moreover, a rapidly evolving field of sustainable nanotechnology requires design guidelines for fabricating sustainable nanomaterials for biomedical applications. Therefore, this review aims to provide an overview of a design framework (i.e., universal design criteria) for synthesizing sustainable nanomaterials based on the data extracted from the original research articles reported in the literature. Later, we discuss how these design guidelines have been applied to produce various sustainable nanomaterials (nanocellulose, carbon, and bioceramic) from renewable resources and their biomedical applications. Finally, we summarize key factors (raw materials, manufacturing process, characterization, and recycling of waste with minimal disposal) that should be taken into consideration for manufacturing sustainable nanomaterials with optimal physiochemical characteristics. By considering these factors, a roadmap for sustainable nanomaterials can be developed with minimal impact on the environment and human.

## 2. Design Framework for Sustainable Nanomaterials

Advances in nanotechnological fabrication approaches have accelerated the discovery of new functional nanomaterials in tunable sizes, morphologies, and composition from non-renewable resources [18,19,20,21]. Such nanomaterials show superior physical and chemical properties compared to their bulk counterpart, making them beneficial to solve societal problems from health to renewable energy, information technology, and the environment [22,23,24,25,26]. However, the long-term commercial viability of these nanomaterials is posing a risk due to their reliance on non-renewable resources and highly energy-intensive manufacturing processes. In addition, there is too little attention paid to investigating the impact of non-sustainable materials on human health, climate change, and the environment. To achieve the SDGs set by the UN, intensive research efforts are needed to design sustainable nanomaterials from renewable resources while optimizing the performance, manufacturing process, and production costs of nanomaterials. Integrating sustainable resources with nanotechnology is critical to building a sustainable society in the 21st century. Sustainability encourages the use of natural resources and lower energy manufacturing methods for designing nanomaterials while maintaining the natural resources for current and future generations. Additionally, the sustainable process prevents waste production (zero waste) or harnesses recycled waste for fabricating nanomaterials to ensure minimal waste disposal, leading towards a circular economy for sustainable development.

When designing sustainable nanomaterials for biomedical applications, natural and renewable resources should be used as precursor materials and surfactants. Nature offers a wide range of various renewable and sustainable materials with rapid renewability, such as biomass and biodegradable natural materials (cellulose, chitosan, lignin) [27,28]. Renewable materials obtained from the photosynthetic process in plants are considered less toxic than synthetic materials. The second most abundant natural material on the planet, known as lignin, is an environmentally friendly material with excellent antioxidant and antimicrobial properties [29]. Cellulose, alginate, chitin, and pectin are other natural polysaccharide molecules that can be derived from microbes, animals, and plants [30]. Nanomaterials derived from natural polysaccharides offer various advantages, such as low processing cost, biodegradability, non-toxicity, and tunable surface functionalities (hydroxyl, amine, and carboxyl groups). Furthermore, nanomaterials can be designed from biomacromolecules (polypeptides, proteins, or nucleic acids) abundant in nature [30]. In addition to natural renewable resources, the waste from chemical industries (e.g., carbon dioxide), agriculture (e.g., rice husk containing ~75–90% organic molecules, such as cellulose and lignin), and the environment (e.g., plastic waste from oceans) can also be considered alternative non-natural renewable resources. This recycled waste from various resources can be used for synthesizing nanomaterials, for example, the use of waste extracted from petrochemicals for fabricating polymeric nanomaterials [31,32].

In bottom-up approaches, surfactants and reducing agents are commonly used to produce nanomaterials via the reduction of precursor materials and stabilize synthesized nanomaterials in the dispersion medium. Natural renewable resources, such as plants, biopolymers, proteins, sugars, bacteria, algae, and fungi, are a few examples that can act as surfactants and reducing agents [33,34,35]. Among various natural resources, biopolymers are the most common reducing agents used in various chemical reactions involved in the bottom-up manufacturing of nanomaterials. Examples of biopolymers that can act as reducing agents include dextran, chitosan, and cellulose extracted from sugarcane, the exoskeleton of crustaceans, and plants. Vitamin C isolated from fruits and vegetables is also a natural reducing agent that can reduce various metal ions in an aqueous solution. However, vitamin C and other natural biomolecules are more expensive than biopolymers. Therefore, these reducing agents are unsuitable for large-scale manufacturing of nanomaterials. The choice of solvent can also be a concern for synthesizing sustainable nanomaterials. Traditional approaches use toxic organic solvents that offer better control over the size, shape, and chemical composition of nanomaterials. The use of such toxic organic solvents should be prohibited or minimized. Water is the most commonly accessible and low-cost solvent for synthesizing sustainable nanomaterials. Alternatively, supercritical fluid technology has received considerable attention in fabricating nanomaterials with control over size, morphology, and composition with minimal environmental impact. This technology uses supercritical fluids, such as water and carbon dioxide (CO_2_), instead of toxic organic solvents [36]. Exploring the use of such alternative strategies will pave the way for the design of sustainable nanomaterials.

Top-down and bottom-up strategies have been developed to fabricate a variety of nanomaterials [37,38,39,40]. However, these energy-intensive approaches use toxic and corrosive precursor and surfactant materials that produce hazardous chemicals or gases. Therefore, the choice of manufacturing process requiring the least energy is essential in fabricating sustainable nanomaterials. The hydrothermal approach is the most popular strategy for synthesizing sustainable nanomaterials [41]. In this approach, the aqueous solution of precursor materials is heated to a high temperature using various heat sources, such as microwave energy and focused sunlight [42,43]. However, these heating mechanisms are not suitable for the large-scale manufacturing of nanomaterials due to insufficient and non-uniform heating of solvents in a large reactor. Recently, cost-effective flow-chemistry-based strategies have been investigated for synthesizing nanomaterials. This method offers better control of heat transfer and reaction mixing time. Another environment-friendly and economically viable approach based on green chemistry has emerged as an alternative strategy to fabricate size- and shape-controlled metallic nanomaterials by using microorganisms (bacteria, fungi, algae, virus) and plant extracts (proteins, polysaccharides, polyphenols) [44,45]. The advantages and disadvantages of physical, chemical, and green synthesis are discussed in the Table 1. Therefore, it is essential to set design guidelines for developing sustainable nanomaterials that can serve as a viable alternative to conventional methods of nanomaterials fabricated from non-renewable sources. Furthermore, the functional performance of sustainable nanomaterials should be optimized in various aspects, for example, renewable materials type (biopolymer, carbonaceous, or composites), physical characteristics (mechanical, thermal, and conductivity), biocompatibility, materials hazard testing, and environmental impact.

## 3. Sustainable Nanomaterials for Biomedical Applications

### 3.1. Sustainable Polymeric Nanomaterials

Polymeric materials have been used for various biomedical applications, including device packaging, tissue engineering, drug delivery, surgical implants, wound dressing, and ophthalmology. In 2021, the global market size for medical-grade polymers was estimated to be USD 18.4 billion, which is expected to increase at an annual growth of 8.9% from 2022 to 2030. Polymers can be classified as synthetic polymers derived from petro-materials and biopolymers derived from biologically renewable resources. The sustainability of the plastics industry is facing rising challenges, such as fossil fuel depletion, the increasing cost of petroleum products, and the long-term catastrophic environmental impact. The fabrication of nanomaterials using renewable biopolymeric materials derived from natural resources is necessary to reduce dependence on petroleum-based plastics. Furthermore, biopolymers derived from natural resources are more biocompatible than polymeric materials derived from petrochemicals.

Among various biopolymers, nanocellulose is a renewable and sustainable nanomaterial derived from native cellulose, the main component of the plant cell [46]. Cellulose is the most abundant biopolymer on the earth, with an annual production of over $7.5\times 10^{10}$ metric tons. It consists of D-anhydro glucopyranose units connected by β-glycosidic bonds, in which the repetitive unit is called cellobiose [47,48]. Cellulose can be extracted from multiple sources, such as plants (e.g., cotton, wood), bacteria, or animals [49,50]. The extraction cost of cellulose from natural resources depends on various factors, such as source of cellulose, extraction method, and scale of production. The plants capture CO_2_ to produce cellulose through photosynthesis and release CO_2_ through degradation, thus closing the carbon cycle. The highly crystallized structure of cellulose provides superior mechanical strength and rigidity to plants. Nanocellulose is produced by the mechanical or chemical breaking of cellulose fibers into their individual nanoscale components. Excellent mechanical strength, light weight, biodegradability, and unique physical properties make nanocellulose useful in applications in fields such as biomedical, packaging, and materials science.

**Figure 2:**
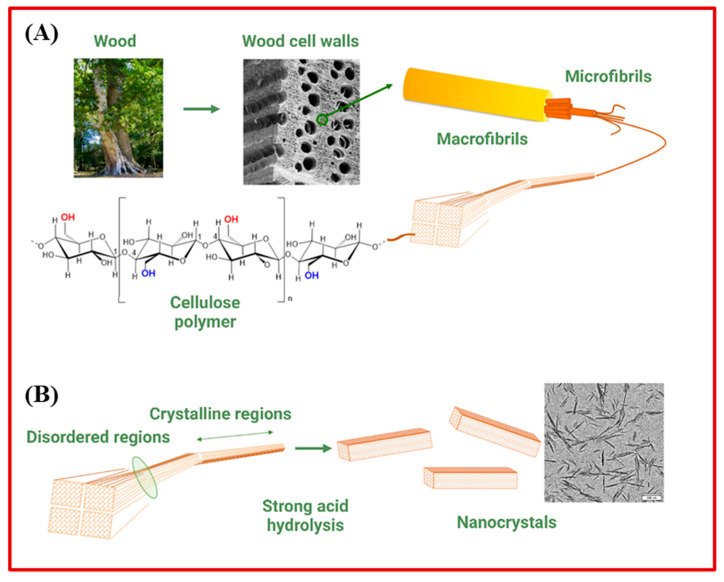
(**A**) The hierarchical structure of cellulose nanofibers derived from the tree. (**B**) The fabrication of cellulose nanocrystals (CNCs) from the nanofibrous network using acid. Adapted with permission from Ref. [57], Copyright 2015, Intech.

**Figure 3:**
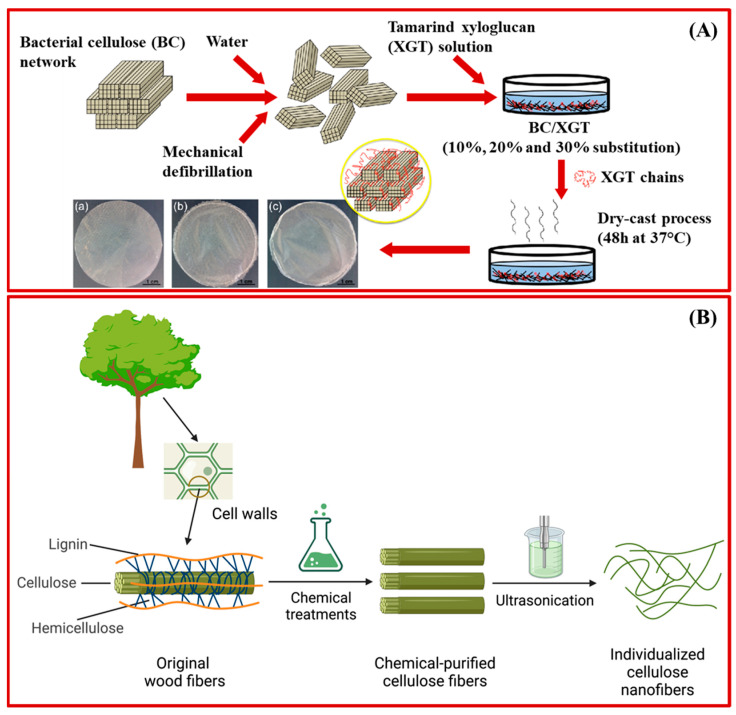
Schematic illustration of (**A**) bacterial nanocellulose (BNC) derived from biocomposite with xyloglucan. Optical images in panel A display the texture of biofilms of (**a**) bacterial cellulose, (**b**) reconstituted bacterial cellulose (RBC) and (**c**) RBC/tamarind xyloglucan biocomposite. (**B**) Schematic drawing of cellulose nanofibers (CNFs) extracted from wood using chemical and ultrasonication procedures. (**A**) is reprinted with permission from Ref. [58] Copyright 2017, Elsevier.

Nanocellulose can be classified into three distinct types based on its morphology and structural characteristics: cellulose nanocrystals (CNCs), cellulose nanofibers (CNFs), and bacterial nanocellulose (BNC). Various top-down approaches have been investigated for fabricating CNCs and CNFs from cellulose. The most common top-down approach for CNCs involves the acidic or enzymatic hydrolysis of natural cellulose derived from wood. This cleaves the amorphous region of the cellulose fibers and facilitates the formation of highly crystalline and rigid nanostructures (Figure 2) [51]. Mechanical decomposition processes, such as ultrasonic fiber delamination, ball-mining, and high-pressure homogenization of biomass, have been explored to synthesize CNFs (Figure 3A). These top-down approaches produce gel-like materials of entangled flexible and short CNF networks [52,53]. Bottom-up approaches have been developed for synthesizing BNC via the metabolization of glucose molecules by Gram-negative *Acetobacter* strains (Figure 3B) [54]. BNC has high chemical purity compared to CNCs and CNFs due to plant impurities, such as lignin and hemicellulose. Therefore, an additional purification step is required to remove the contaminants [55]. Despite different strategies used for fabricating CNCs, CNFs, and BNC, all of these nanocellulose materials possess identical molecular structures to cellulose, but they have a larger surface area, stiffness, and aspect ratio than cellulose. Moreover, nanocellulose possesses superior mechanical strength, biocompatibility, biodegradability, chemical stability, and sustainable abundance on earth, thus making these nanomaterials suitable for biomedical applications [56]. Table 2 summarizes cellulose materials (CNCs, BNCs, and CNFs) obtained from various natural resources and their biomedical applications.

BNC has a unique nanofibril network with a large surface area that mimics an extracellular matrix. It has an exceptional capability to absorb exudates from wounds, strong water retention ability, excellent conformability, and wet strength [51]. Therefore, BNC can be used for wound dressing. Few BNC-based products, such as XCell^®^ and Biofill^®^, have been commercialized for wound healing [55]. Compared to traditional cotton-based hemostatic wound dressings, nanocellulose can be functionalized to promote wound healing and prevent secondary infection. Antimicrobial agents such as benzalkonium chloride, silver, and copper nanoparticles can be impregnated into the porous network of BNC’s membrane that acts as a physical barrier between the wound and the surrounding environment and facilitates the steady release of preloaded antimicrobials [59,60,61,62,63]. Furthermore, BNC can be integrated with other naturally derived materials to design next generation innovative sustainable nanomaterials. For example, integrating other natural biopolymers (chitosan and hyaluronan (HA)) into BNC can provide additional functionalities during wound healing because chitosan possesses excellent antimicrobial properties [64], and HA facilitates rapid wound healing and reduce scar tissue formation [65].

**Table 2:** Summary of nanocellulose material for biomedical applications.

Sustainable Nanomaterials	Natural Source	Biomedical Application
Cellulose Nanocrystals (CNCs)	Wood, cotton, and other cellulose-rich sources	Antimicrobial purpose [59,60,61,66] Bioimaging (osteoblasts cellular imaging [67]) Biosensing (DNA sensing [68]) Tissue engineering (fibroblasts proliferation [69]) Drug delivery [70,71]
Cellulose Nanofibers (CNFs)		Antimicrobial purpose [72,73,74] Tissue engineering scaffold (bone [75], ligament and tendon [29]) Ion-exchange membrane (DNA extraction and hemodialysis [76]) Reinforcing agent [77] Drug delivery [78]
Bacterial Nanocellulose (BNC)	Glucose molecule	Functionalized wound dressing (antimicrobial [64,65]) Soft tissue engineering (cartilage, [79,80], bone [81], vessels [79])

Tissue engineering holds great promise to utilize biocompatible materials (cells and biomolecules) to repair and restore damaged tissues. Therefore, it is vital to design materials/scaffolds mimicking the native tissue-like environment to promote cellular activities (adhesion and growth) and tissue growth. Such platforms can be developed by incorporating nanofibrils of BNC into hydrogel with superior mechanical strength, biocompatibility, and biodegradability [79,82]. Nimeskern et al. developed BNC–hydrogel platforms that have similar mechanical moduli of human cartilage [79]. They showed that these composite materials can be transformed to complex patient-specific shapes and geometries. Apelgern et al. developed an aqueous counter-collision method to disassemble BNC to create a bioink for cartilage-specific 3D bioprinting. In vivo studies demonstrated that the cell-laden BNC structures had good structural and tissue integrity, and excellent chondrocyte proliferation, making BNC suitable for cartilage regeneration [80]. Cañas-Gutiérrez et al. fabricated 3D-printed BNC scaffolds with controlled microporosity between 50 and 350 µm and demonstrated the adherence and proliferation of osteoblasts on a 3D-printed scaffold for bone regeneration [81]. In the work of Backdahel et al., it was found that BNC had a similar stress–strain response to the carotid arteries, thus supporting human smooth muscle cell attachment and proliferation after 14 days of culture [79]. BNC can be transformed into various structures due to its excellent moldability. Therefore, such materials can also be used for designing small-caliber artificial blood vessels. However, modification with anticoagulant agents (heparin) and chimeric proteins (cellulose-binding module and cell adhesion peptides) are needed to improve the properties of BNC in vascular grafts [81,83].

CNFs exhibit non-toxicity to humans and the environment [84,85,86]. Since CNFs also possess a large surface area and surface density, composite materials with antimicrobial characteristics have been designed by incorporating metallic nanoparticles (NPs), such as Ag NPs [72], ZnO NPs [73], and TiO_2_ NPs [74]. CNFs have been used as cell culturing scaffolds for tissue engineering applications due to their excellent mechanical strength, ultralow density, and biodegradability. Carlström et al. blended and cross-linked gelatin with wood-derived CNF scaffolds to adjust the degradation time and loaded human bone marrow mesenchymal stem cells into the scaffold [75]. They showed the applicability of CNF scaffolds for supporting cell attachment, spreading, and osteogenic differentiation. In another work, Mathew et al. used a CNF scaffold for ligament and tendon regeneration [29]. CNFs have also been used as an electrochemically controlled ion exchange material due to their large surface area and high mechanical strength [87]. Mihranyan et al. coated a thin layer of polypyrrole (PPy) onto CNF paper to fabricate PPy–CNF composites for DNA extraction and hemodialysis membranes [76]. The use of CNFs as a reinforcing agent for improving the mechanical properties of the hydrogel system has been demonstrated by Maharjan and co-workers. They incorporated CNFs into the chitosan hydrogels to fabricate a CNFs/Chitosan scaffold with a small pore size of uniform distribution. The hybrid system showed increased compressive strength (30.19 kPa) compared to the pure chitosan scaffold (11.21 kPa) and enhanced bioactivity of the scaffold [77]. Furthermore, CNF exhibited strong molecular interaction between poorly soluble drugs and drug-encapsulated nanoparticles with tailored drug release kinetics, making them a promising candidate for drug delivery applications [78].

Like BNC and CNFs, CNCs also possess excellent biocompatibility and mechanical properties. Inorganic NPs (Au, Ag, and Pd) can be incorporated into CNCs to add new functionalities, such as antimicrobial properties, bioimaging, and biosensing capabilities [66]. Ganguly et al. developed an eco-friendly diagnostic tool for detecting unamplified pathogenic DNA using TEMPO-oxidized CNC-capped gold nanoparticles. A dramatic color shift from red to blue was observed in the presence of target DNA molecules [68]. Rueda et al. designed mechanically strong and ductile polyurethane/CNC nanocomposites via in situ polymerization to support the proliferation of fibroblasts [69]. CNCs can also be used as carriers to deliver bioactive drug molecules to the target site because the hydrophilic surface can inhibit the formation of protein corona on CNCs, thus prolonging the CNC half-life in the bloodstream [70]. Seo et al. developed multilayer CNCs for anticancer drug delivery by coating the negatively charged CNCs with cationic doxorubicin (DOX) and anionic hyaluronic acid (HA) polymer as the tumor-targeting ligand. The nanocomposite system showed excellent tumor penetration, cellular uptake, and cancer-killing ability through intravenous injection [71]. To design a bioimaging probe, fluorescein (isothiocyanate (FITC)) can be conjugated onto the CNC surface. The developed FTIC–CNC probe showed high fluorescence intensity and cellular uptake, no cellular toxicity to mouse osteoblasts, and enhanced dispersity in the biopolymer matrix [67].

Despite the great promise of nanocellulose, the economic development of nanocellulose-based biopolymers poses a significant challenge. A balance between the use of crops for food, environmental protection, and the production of raw cellulose materials is required to achieve sustainability in nanocellulose production for future generations. Research efforts have been commenced to genetically modify plants with the ability to produce biopolymers in plant stems. Concurrently, the energy needed to produce and modify the nanocellulose from its original state must be considered to curb the total amount of carbon emission.

### 3.2. Carbonaceous Sustainable Nanomaterials

**Figure 4:**
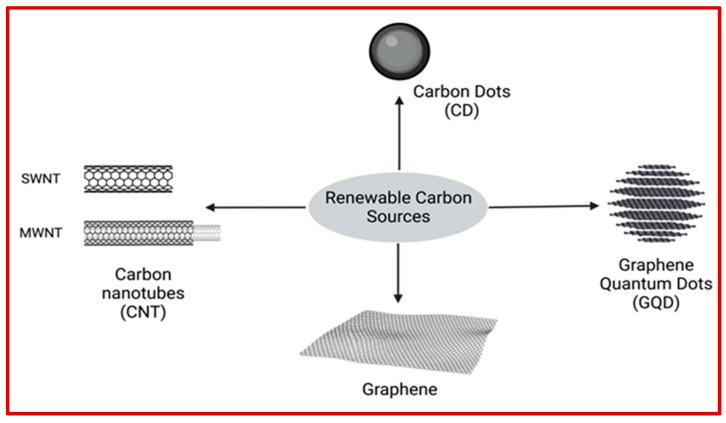
Sustainable production of different carbonaceous nanomaterials (CNMs) from renewable carbon resources.

Carbon is the most abundant non-metal element that appears naturally in various forms, such as coal, diamond, and graphite [88]. Carbonaceous nanomaterials (CNMs) are carbon-based materials designed to reduce environmental impact while retaining their desirable properties. Such materials can be classified based on the number of dimensions. Carbon dots (CDs) and graphene quantum dots (GQDs) are classified as zero-dimensional CNMs. Carbon nanotubes (CNTs), graphene, and diamonds are examples of one, two, and three-dimensional CNMs, respectively. CDs, GQDs, CNTs, and graphene are the most common CNMs with exceptional properties, which make them suitable for various biomedical applications, from biosensing to diagnosis, targeted drug delivery, bioimaging, and tissue engineering. Compared to other nanomaterials such as metal or organic nanoparticles, CNMs with large surface area to volume ratios offer several advantages such as higher drug-loading capacity, higher biocompatibility, straightforward surface functionalization, easier immobilization of macromolecules, and excellent physical properties (electrical, optical, and thermal conductivity) [89,90,91]. Even though conventional strategies have been developed to scale-up manufacturing of CNMs using traditional methods, these strategies are expensive due to the use of energy-intensive processes. In recent years, the focus of research in the field of CNMs has shifted to fabricating CNMs using less energy-intensive methods from sustainable and renewable materials. Biomass and waste residue (derived from industrial effluents, plastics, and agriculture) are good candidates for the sustainable production of CNMs because of their rich geographic availability on earth (Figure 4). The extraction cost of carbonaceous materials from sustainable resources can be varied from few hundreds to several thousand USD per year depending on the type of sustainable resources, synthetic methodologies, and purification process. Table 3 summarizes carbonaceous nanomaterials obtained from natural and sustainable resources and their biomedical applications.

#### 3.2.1. Carbon Dots and Graphene Quantum Dots

Carbon dots (CDs) are quasi-spherical carbon nanoparticles composed of C, H, N, and O with sizes smaller than 10 nm [92]. The macroscopic carbon has low water solubility, but the presence of hydrophilic carboxylic and amine groups on the CDs’ surface makes them well dispersed in water. Graphene quantum dots (GQDs) are crystallized graphene disks usually composed of less than 10 graphene layers with a size between 2 and 20 nm [93]. Both CDs and GQDs show strong photoluminescence emission within the visible light spectrum, high photostability, and resistance to photobleaching [94]. The excellent biocompatibility and ease of surface modification due to the presence of hydrophilic groups make them suitable for biomedical applications.

The traditional approaches for CDs synthesis, such as laser ablation, electrochemical method, pyrolysis, and exfoliation, require expensive initial precursor non-renewable materials, longer reaction times, and harsh reaction environments [95]. In recent years, sustainable approaches have been developed for synthesizing CDs and GQDs using sustainable and renewable sources, such as organic pollutants available in the environment [96,97] and various biomass (vegetables, fruits, wool, cotton) [98]. In order to make manufacturing process less energy intensive, Xu et al. modified the traditional laser method by adopting a femtosecond pulsed laser of low power density for synthesizing CDs at room temperature [99]. Menezes et al. developed a green electrochemical method to produce GQDs using platinum wire as a cathode and a graphite rod as an anode without using toxic chemicals (oxidant salts and acids) [100]. A sustainable and simple one-step hydrothermal carbonization method has been developed to synthesize CDs from oyster mushrooms for selective sensing of Pb^2+^ ions, which show specific electrostatic binding to DNA molecules, antibacterial activity, and anticancer activity against MDA-MD-231 breast cancer cells [101]. In the work of Sangam et al., a catalyst-free and scalable hydrothermal method has been used for synthesizing GQDs from agro-industrial waste sugarcane molasses [102].

**Table 3:** Summary of carbonaceous material for biomedical applications.

Sustainable Nanomaterials	Sustainable Source	Biomedical Application
Carbon dots (CDs)	Organic pollutants/wastes, vegetables, fruits, woolds, cottons, oyster mushroom	Biosensing (DNA [101,103,104,105], protein and disease-related biomolecules [106]) Cellular bioimaging (bone regeneration monitoring [107]) Drug delivery [108]
Graphene quantum dots (GDs)		Biosensing (DNA, protein, disease-related biomolecules) [109] Cellular bioimaging [110] Drug delivery [108]
Graphene	Organic carbonaceous wastes (bone, bagasse newspapers), plants, and natural	Limited due to aggregation in tissue
Graphene oxide (GOX)		Drug delivery [111] and transdermal drug delivery [112] Microelectrodes [113] Biosensing and tissue engineering
Carbon nanotubes (CNTs)	plants (leaves, seeds, roots, and stem), waste cooking oil	Electromechanical actuators [114] Biosensors (miRNA [115]) Cellular bioimaging [116] Drug delivery [117] Photothermal cancer therapy [118] Nano tweezers [119,120]

CDs and GQDs have extensively been used to develop inexpensive biosensors for rapidly determining analytic concentration with picomolar detection sensitivity in biological samples. CDs have been used for the selective sensing of DNA [103,104,105], protein, and other blood serum components such as cholesterol, glucose, and alcohol [106]. Similarly, GQDs have been used for the sensitive detection of different disease biomarkers such as enzymes, antigens, proteins, DNA, and other biomolecules [109]. In addition to biosensing, CDs and GQDs have been developed for bioimaging applications due to their excellent biocompatibility, high photostability, and high fluorescent intensity [121]. Saranti et al. incorporated CDs into bioactive scaffolds to monitor the bone regeneration process through bioimaging [107]. In another work, Gong et al. synthesized N and Br co-doped GQDs using a rapid and large-scale approach. The results showed high cellular uptake and high-quality fluorescence labeling ability, suggesting their great promise for cellular bioimaging [110]. Since these nanomaterials have hydrophilic surface groups (carboxylic and amine), the covalent or non-covalent surface modification strategies can be used to attach targeting and drug molecules on the surface of CDs and GQDs for designing targeted drug delivery platforms for the treatment of cancerous diseases [108].

#### 3.2.2. Graphene

Graphene is a two-dimensional CNM that consists of a single layer of densely packed $sp^{2}$ carbon atoms arranged in a hexagonal structure [122]. Graphene exhibits unique physical properties compared to the bulk structure, including large surface area, high electrical and thermal conductivity, high elasticity and mechanical strength, and tunable optical properties [123]. Graphene oxide (GOX) is chemically functionalized graphene with a surface modified with oxygen-containing groups (carboxy, carbonyl, and hydroxyl). The presence of oxygen-containing groups on the surface enhances the hydrophilicity of the graphene, which contributes to the aqueous solution stability of graphene due to stronger repulsive interactions, such as electrostatic and hydrogen bonding with other molecules besides π−π interaction [124]. Therefore, graphene, especially GOX, has been widely investigated for various biomedical applications. Conventional strategies, such as mechanical or chemical exfoliation of graphite, have been developed for graphene production. However, these methods require toxic and expensive chemical reagents to purify graphene, restricting their ability to scale up production [125]. Alternative strategies have been researched to fabricate graphene from sustainable and renewable materials, including industrial waste, food waste, plants, agriculture waste, and natural carbonaceous wastes (e.g., timber, bagasse, animal bones, and newspapers) through graphitization [126]. Wei et al. developed an aerogel composite using bacterial cellulose and caffeic acid-reduced graphene oxide for designing a bio-pressure sensing-based wearable device [127]. In the work of Somanathan et al., an environmentally friendly strategy has been established for synthesizing GOX via single-step reforming of sugarcane bagasse waste under atmospheric conditions [128].

The one-atom-thick carbon layer provides a high surface area for binding drug molecules and targeting ligands to both sides of graphene [129]. Therefore, graphene-based nanomaterials show enormous potential for drug delivery with higher drug-loading efficiency. However, graphene can aggregate in tissues, which may generate oxidative stress and causes toxic effects on humans [122]. The aggregation issue can be overcome by employing surface modification strategies. Prabakaran et al. functionalized GOX with ovalbumin protein and polymethyl methacrylate through a simple chemical reaction, resulting in the fabrication of a stable and biocompatible composite system [111]. In another work, the incorporation of GOX into the polymeric microneedle-based transdermal drug delivery system has also been shown for delivering anti-melanoma chemotherapeutic HA15 molecules. The results showed significant enhancement in the mechanical strength and moisture resistance of the device fabricated by incorporating GOX into polymeric materials, providing anti-inflammatory properties [112]. Lu et al. developed a flexible cortical microelectrodes array using porous graphene. Superior durability, mechanical strength, impedance, and charge injection properties make these graphene microelectrode arrays suitable for deep brain signal sensing and stimulation [113]. The potential of functionalized graphene has been explored for biosensing [130,131] and tissue engineering applications [132,133].

#### 3.2.3. Carbon Nanotubes

Carbon nanotubes (CNT) are made of a layer of graphene that forms a cylindrical structure with dimensions in nanometers [119]. Depending on the graphene cylinder arrangements, CNTs can be classified as single-walled carbon nanotubes (SWCNTs) and multilayer-walled carbon nanotubes (MWCNTs). CNTs show unique physical properties, such as superior thermal conductivity, high electrical conductivity, and high mechanical strength. While significant progress has been made in the scalable manufacturing of CNTs, traditional methods such as chemical vapor deposition, electric arc discharge, and spray pyrolysis rely on high temperature, low pressure, non-renewable materials, and toxic solvents. These drawbacks can have negative environmental and health impacts [95,134]. Therefore, alternative strategies are needed for fabricating CNTs by utilizing renewable resources. Catalytic chemical vapor deposition is one of the most common strategies to produce CNTs. Using bioderived precursor materials can make the chemical vapor deposition process less energy-intensive (i.e., reducing the reaction time and temperature) [135,136]. Green synthesis methods have been developed to produce CNTs using renewable resources such as plants (leaves, seeds, roots, and stems) [134]. Duarte et al. synthesized CNTs from waste cooking oil using the CVD method [137]. Microwaves can be considered an environment-friendly approach that utilizes electromagnetic energy to heat precursor materials [131]. This less energy-intensive approach has enabled the rapid production of CNTs from various carbon sources, catalysts, and substrates [138].

CNTs can be used as electromechanical actuators when the potential is applied to an electrolyte. Ru et al. used a nanoporous CNTs film as the electrode for ionic electroactive polymer actuators. The electrode showed superior conductivity and improved electromechanical and electrochemical properties with enhanced durability under various voltages and frequency ranges, making them suitable for artificial muscle application [114]. CNTs have been used to design optical or electronic biosensors for biomolecular detection, such as DNA, glucose, and proteins. Li et al. developed a field-transistor biosensor using polymer-sorted semiconducting CNT films to detect exosomal miRNA with high sensitivity for breast cancer detection [115]. Because of photoluminescence, Raman scattering, photoacoustic, and echogenic properties, CNTs have been investigated for tracking and bioimaging in biological environments [119]. Singh et al. fabricated MWCNTs by the pyrolysis of a chickpea peel precursor. These MWCNTs showed blue fluorescence signals in human prostate carcinoma cells without cytotoxicity [116]. The application of CNTs has been demonstrated to deliver drugs of low solubility and low bioavailability to the target site via enhanced cellular permeation [117]. Suo et al. functionalized MWCNTs with a layer of the phospholipid-poly(ethylene glycol) and anti-Pgp antibodies to improve the biocompatibility, blood circulation, and ability to target cancer cells. In this work, they showed the effectiveness of the phospholipid–PEG-coated MWCNTs functionalized with anti-Pgp antibodies in generating phototoxicity in cancer cells under photoirradiation without damaging normal cells [118]. CNTs can be used as flexible nanotweezers, facilitating simultaneous changes and variations in biomedical analytical studies, such as nucleic acid based spectroscopy [119,120].

### 3.3. Sustainable Bioceramics

Bioceramic is an important biocompatible ceramic material with excellent bioactivity, chemical stability, thermal resistance, and tissue-like mechanical characteristics. Bioceramics have been used as a nanoporous scaffold material to repair and reconstruct damaged tissues, fill bone defects, and deliver drug molecules [139,140,141,142]. Bioceramics, such as calcium phosphate and hydroxyapatite, can be extracted from natural waste materials (eggshell, bovine bone, fish bone, and seashells), which are widely geographically available at low cost. Since these natural raw materials are derived from sustainable biological systems, they do not possess the inherent toxicity or potential side effects on exposure often shown by synthetic materials. For example, eggshells are an abundant biowaste from food processing industries, produced at an annual rate of approximately 250,000 tons per year. This is ranked as the 15th most common pollutant by the Environment Protection Agency [143]. Given the increased egg consumption worldwide and enrichment with minerals (calcium carbonate, calcium phosphate, 1% magnesium), eggshell biowaste can be used for synthesizing bioceramics, including calcium phosphate and hydroxyapatite. Furthermore, eggshells contain a trace amount of biologically relevant ions (Mg, Na, Si, and Sr), which facilitates the mineralization process and promotes bone growth.

Calcium phosphate (CaP) is the most widely researched synthetic biomaterial used in bone regeneration because of its natural presence in the human bone, superior bioactivity, biocompatibility, and biodegradability. CaP can be classified as α- or β-tricalcium phosphate (α- or β-TCP), biphasic calcium phosphate (BCPs), and calcium hydroxyapatite. Various synthesis methods, such as sol–gel, hydrothermal, solid-state reaction, ultrasonic, and microwave, have been investigated to fabricate CaP biomaterials from eggshell biowaste. For example, a wet chemical method followed by heating at high temperatures and ball milling has been explored to synthesize crystalline β-TCP biomaterials derived from the eggshell [144,145]. Hydroxyapatite materials have been produced by the solid-state decomposition of eggshells at elevated temperature (1050 °C) [146]. Among various synthetic strategies, microwave synthesis is the most environment-friendly approach for manufacturing bioceramic nanoparticles with narrow size distribution at a high throughput. The size, shape, and crystallinity of CaP bioceramics can be tuned by varying the microwave parameters, such as microwave power and exposure time [147,148]. Bioceramics derived from eggshells have been shown to exhibit superior biological performance compared to synthetic bioceramics. This is because the eggshells are formed from a complex matrix of calcium carbonate, calcium phosphate, and organic proteins, which contributes to their unique mechanical and biological characteristics. Kumar et al. developed an efficient protein delivery system using natural and sustainable materials. The in vitro results showed improved encapsulation efficiency of protein cargo and enhanced protein delivery compared to synthetic TCP with a similar Ca/P ratio [149]. Hydroxyapatite and β-TCP bioceramics have been investigated as bone graft substitutes due to their excellent biocompatibility, bioactivity, and osteoconductivity. Sangjin et al. demonstrated that scaffolds made from eggshell-derived hydroxyapatite and β-TCP were effective in promoting bone formation in rabbits [150]. The superior biological performance of eggshell-derived materials resulted from the presence of biologically relevant ions in the eggshells. Therefore, sustainable bioceramics hold great potential to address the biomedical challenges posed by synthetic bioceramics. However, there is a need for scalable and continuous manufacturing of bioceramics extracted from eggshells, which requires a government policy regarding the collection of eggshell biowaste and supply to the industry manufacturers and research organizations.

## 4. Conclusions and Future Opportunities

The concept of sustainability has been coined to address the societal challenges in the energy, environment, economy, and biomedical fields, supporting the UN’s SDGs for present and future generations. This review summarizes the main design principles for fabricating sustainable nanomaterials using renewable resources and environment-friendly scalable manufacturing methods with minimal hazardous waste generation (zero-waste). The use of recycled waste as a renewable resource for fabricating sustainable nanomaterials is a promising strategy towards achieving a zero-waste manufacturing process. By doing so, we can achieve the circular economy model and responsible waste management practices contributing to sustainability. We also discussed sustainable nanomaterial design principles that have been applied to fabricate nanocellulose, carbon, and bioceramic from renewable resources, such as plants, woods, fruits, biopolymers, bacteria, eggshell bio-waste, and recycled waste. The sustainable nanomaterials derived from natural resources offer several benefits over those nanomaterials produced from non-renewable resources, such as versatile surface functionalities, biocompatibility, and biodegradability.

Renewable resources are endless. However, the performance of sustainable nanomaterials produced from renewable resources is still inferior to nanomaterials synthesized from non-renewable resources. In addition, questions concerning the manufacturing process optimization (cost and geographic availability of natural resources) and their actual impact on human health, climate, and the environment are yet to be addressed. There are lessons to be learned from the rapid innovation in nanomaterials production from non-renewable materials, where the high performance of functional materials was prioritized without considering the impact of raw materials’ future availability, energy resources, and toxic chemicals on the environment and human health. Moreover, the entire life cycle of nanomaterials, from production to disposal has not been considered. Therefore, there is a need to develop responsible nanomaterials manufacturing practices considering the appropriate selection of renewable materials based on inherent functionality, geographic availability, and cost.

There are several areas where future research in sustainable nanomaterials could be directed. First, the development of environmentally friendly strategies based on green chemistry for synthesizing sustainable nanomaterials is an essential step towards creating an eco-friendly sustainable future. The use of natural resources with rapid renewability should be prioritized. Second, the performance of sustainable nanomaterials should be optimized with respect to appropriate renewable resources (resource type, extraction cost, and geographic availability) and manufacturing methods (energy requirement, waste production, and scalability). The renewable material selection should be justified based on the energy requirement for manufacturing, potential waste production, material source (renewable or non-renewable), biocompatibility, biodegradability, and recyclability. Third, the performance of sustainable materials should be optimized in the intended targeted environment (i.e., optimization of physiochemical properties in a biological environment for establishing the relationship between the nanomaterial design and properties). Fourth, a comprehensive assessment of the environmental and human health impact of nanomaterials throughout their entire life cycle, from production to disposal, should be conducted. Fifth, new sustainable methods for the recycling and disposal of nanomaterials should be encouraged. Finally, new regulations and standards should be established for producing and using nanomaterials that prioritize sustainability and minimize harm to human health and the environment.

Furthermore, a sustainable nanomaterial performance matrix analog to Ashby’s materials selection should be set [151]. This performance matrix can include various parameters, such as the behavior of nanomaterials in biological fluid (because the presence of biomolecules or proteins can alter the physiochemical properties), optimization of nanomaterials’ performance against intended biomedical applications, and long-term toxicity of nanomaterials. The environmental risk associated with developed nanomaterials can also be included in the performance matrix because releasing nanomaterials into the environment can contaminate the land and groundwater (i.e., decreasing crop productivity and water quality). Developing a performance matrix of sustainable nanomaterials will generate an extensive dataset that can be analyzed using machine learning. In the future, machine-learning-based approaches will facilitate the informed design of sustainable nanomaterials with an optimal performance by analyzing large datasets [152]. This sustainable materials design framework can bridge the gap between research and commercialization. We expect that natural and sustainable resources offer an opportunity for fabricating next generation sustainable nanomaterials using a less energy-intensive eco-friendly method without causing harm to human health and the environment. New nanomaterials design practices will also contribute to the SDGs of the UN and the circular economy model.

## Figures and Tables

**Table 1:** Advantages and disadvantages of physical, chemical, and green synthesis.

Physical Methods	Chemical Methods	Green Methods
**Advantages**		
Production of nanomaterials with control over size and shape	Synthesis of nanomaterials from a range of materials	Sustainable and environmentally friendly methods
Fabricating nanomaterials with high purity	Control over size, shape, crystallinity, and surface functionality	Fabrication of biocompatible nanomaterials from non-toxic and renewable resources
Scalable	High throughput fabrication of nanomaterials	Less energy intensive method with minimal waste
**Disadvantages**		
Fabrication of nanomaterials from limited materials	Energy intensive	Limited scalability
Energy intensive and expensive	Require toxic chemicals and generate hazardous waste as reaction byproduct	Limited control over size, shape, and physical properties
Some physical methods (e.g., etching) require toxic gases and chemicals	Require additional steps of modification to improve biocompatibility	Restricted to the fabrication of nanomaterials from few materials
Not eco-friendly	Not eco-friendly	Reproducibility
